# Evaluation of Spanish Health System during the COVID-19 Pandemic: Accountability and Wellbeing Results

**DOI:** 10.3390/ijerph182412907

**Published:** 2021-12-07

**Authors:** José Antonio Peña-Ramos, Fátima Recuero-López, Antonio Sánchez-Bayón, Francisco Javier Sastre

**Affiliations:** 1 Faculty of Social Sciences and Humanities, Universidad Autónoma de Chile, Providencia 7500912, Chile; 2 Faculty of Political Sciences and Sociology, Universidad of Granada, 18071 Granada, Spain; frecuero@ugr.es; 3 Department of Business Economics (ADO), Applied Economics II and Fundamentals of Economic Analysis, Legal and Social Sciences School, Universidad Rey Juan Carlos, 28033 Madrid, Spain; 4 ESIC Business & Marketing School, 28224 Pozuelo de Alarcón, Spain; franciscojavier.sastre@esic.edu

**Keywords:** health system, health management, transparency, communication, reputation, well-being, COVID-19, Spain, B5, D6, H5, I1, K3, P16, Z1

## Abstract

The COVID-19 pandemic poses a challenge for health systems. For this reason, it is essential to evaluate the management of health systems in the face of the pandemic, identifying the factors that may contribute to its failure or success. This management is more difficult in decentralized countries, since in them, health competencies are distributed among different levels of government. This is the case in Spain, one of the countries most affected by the pandemic. Therefore, the aim of this article is to evaluate how the Spanish health system has managed the COVID-19 pandemic. Four factors related to health management are analyzed: transparency, communication, reputation and well-being generated. For this purpose, a quantitative analysis is used with the contrast of secondary sources, such as the Merco rankings or survey data from the Centro de Investigaciones Sociológicas (Sociological Research Center). The results show that although the flow of communication about the health system increases considerably, such information comes mainly from the media, with a deficit in the transparency of health management. Likewise, although the reputation of the health system increases at the beginning of the pandemic, as it progresses, there is a deterioration in citizen satisfaction with the healthcare management and the services provided, as well as in the well-being generated by them. This study may have implications for decision making by public authorities regarding the different factors of health management.

## 1. Introduction

A new coronavirus, named SARS-CoV-2, appeared in China at the end of 2019. This coronavirus generated a new disease, COVID-19, which had major consequences for human health, becoming a pandemic in March 2020 as it spread rapidly throughout the rest of the planet. The rapid increase in infections, hospital admissions and deaths has put the health systems of all countries of the world under pressure [1].

Health systems are on the front line in the fight against the pandemic, being the center of the response to it [2]. This has been an important test for health systems around the world, showing the capacity that each of them has to face an unforeseen health emergency of great magnitude [3]. In this sense, health management becomes essential to mitigate the consequences of COVID-19 among the population. The management carried out in each country shows the experience of failures and successes in dealing with the virus.

The perspective provided by the time elapsed since the start of the health crisis allows us to examine the response. Detecting the factors that contribute to failure or success in the fight against the pandemic is essential while we are still immersed in it. Likewise, it is an experience that allows conclusions to be drawn for the improvement of health management and for the strengthening of health systems in the face of future health emergencies [4].

Some studies have had the opportunity to examine the factors that have contributed negatively or positively to the management of the pandemic in different countries. It is necessary to point out that the COVID-19 pandemic has been a challenge that has generated great difficulties throughout the planet. However, the different health management strategies implemented by the health authorities, together with the preconditions of each health system, contribute to explaining the worst or best results obtained in each place.

Many health systems have experienced major problems, being on the verge of collapse. This situation has been favored by factors such as the deterioration that some of them have suffered in recent years. Cuts in economic resources, the privatization of services or the deterioration of the working conditions of health personnel have put many health systems in a precarious starting position in the face of COVID-19. The subsequent development of the pandemic worsens this situation in the face of increasing hospital pressure with the rapid spread of infections. This meant a deterioration in the quality of care, the impossibility of attending to all patients (scarcity dilemma) and the neglect of patients with pathologies other than COVID-19, which will put greater pressure on health systems in the future [5,6,7].

The territorial decentralization of health policy and the fragmentation of the health structure and services would be factors that would make difficult the fight against the pandemic. In decentralized countries, health management is distributed among different levels of government, making the fight against the pandemic more complex. The regions usually have their own health systems, with the central government maintaining coordination powers to a greater or lesser extent. This makes it difficult to convey a clear message to stakeholders on how to act to deal with COVID-19. Countries that have a decentralized health system have tended to show greater difficulties in managing the pandemic. This would be reflected, for example, in the cases of Italy and the United States. The fragmentation of health management would have generated inefficiency and inequality in the services that citizens receive. Thus, greater national coordination would be necessary [8,9].

The success cases in the management of the coronavirus, meanwhile, show a series of common factors. In line with the above, collaboration is presented as essential. Such collaboration occurs not only between institutions and levels of government, but also together with civil society, the media and private individuals. This would be the case, for example, in Vietnam, South Korea and Kerala. The involvement of all stakeholders, that is, of the entire society, shows greater efficiency in reducing infections and managing their consequences [2,3,4,5,6,7,8,9,10].

This leads us to the importance of governance, it being necessary that there be a clear distribution of functions and responsibilities among the different actors, especially between the institutions. The confusion in this distribution of responsibility damaged the management in various countries, especially the decentralized ones [4].

The management of these functions and responsibilities by the health authorities involves the provision of services to citizens. During the pandemic, services such as testing, case detection or contact tracing of positive cases have been essential in the management of COVID-19 [4]. The evaluation of this health management by the different stakeholders shapes the reputation of the health system. This reputation is related to the legitimacy of the health management [11].

Health authorities “are responsible for carrying out a range of services to ensure population well-being” [4] (p. 266). In this way, health management has a direct impact on the well-being of citizens. Thus, the satisfaction of citizens with the health system and health management is an important element for their evaluation, as they are the ones who experience first-hand the success or failure of the configuration of the health system and the management carried out.

Transparency is also a factor that accompanies the successful management of the pandemic. The publication of all the available information by the health authorities allows better coping with COVID-19 and the accountability of health management [4,5,6,7,8,9,10,11,12]. Likewise, transparency would be a key element to favor cooperation and trust between health institutions and the different stakeholders in society [13].

However, it is not enough for the information to be available; it is necessary for the information to reach citizens for it to be effective. Communication, therefore, is an indispensable factor in a health crisis. The successful cases of pandemic management show a firm commitment to communication. The public disclosure of information that allows citizens to know the rules to stop infections, and what to do after contracting the disease, contributes to reducing the transmission of it. The alliance of the health authorities with the media in this task is essential for the message to be clear, avoiding misinformation, and for its dissemination. Collaboration between actors, again, is shown to be fundamental. Digital technologies have played a key role in communication, as well as in maintaining social distancing measures [14,15,16,17].

In this context, in which factors that can harm and favor the fight against the pandemic are identified, the case of Spain is of interest. Spain was one of the countries most affected by the pandemic at the beginning of it. Spain faces the important challenge of COVID-19 in a context in which health management is decentralized in the regions, with the national government maintaining basic and coordination competencies. This distribution of power has generated conflicts over which level of government, national or regional, is responsible for approving public policies in the face of the pandemic, so there is no clear distribution of functions and responsibilities. 

In addition to this, the Spanish health system is facing the pandemic crisis with a precarious starting situation, due to cost containment [18] and the precariousness of health personnel [19], among other things, and structural problems [20]. The decentralization of the health system would also generate different scenarios among the Autonomous Regions, since each of them would face the pandemic with different resources [21]. This would be compounded by the scarcity of the necessary tools to fight COVID-19 at the initial stage of the pandemic, such as respirators, PPE, masks or gloves; and the wear and tear on the health system, especially on human resources, in later phases of the pandemic [22,23].

Therefore, it is necessary to examine the Spanish health management against COVID-19, taking into account its regional decentralization, focusing on the factors that contribute to success in the fight against the pandemic. According to that, this study is based on the following research question: How has the Spanish health system managed the COVID-19 pandemic? The objective is, therefore, to evaluate the Spanish health system during the COVID-19 pandemic.

To this end, first, the Spanish health system is described, deepening its decentralized nature. Second, the factors through which the health system is evaluated are presented. Third, the working hypotheses of the study are presented below. Fourth, the data and the methodology used to test the hypotheses are detailed. Fifth, the results obtained on the evaluation of the Spanish health system during the pandemic are presented. Sixth, the findings obtained are discussed; and, finally, the conclusions reached are presented.

### 1.1. The Spanish Health System

In Spain, the political–legal system is a semi-federal model, divided into seventeen Autonomous Communities (ACs) and two Autonomous Cities [24], where the healthcare system is decentralized and transferred in several phases. The first step was the General Health Act of 1986, although the responsibility for health had already been transferred to Catalonia in 1981, followed by seven more Autonomous Communities and, finally, in 2001, it was extended to all of them. This means the coexistence of seventeen health systems, one for each Autonomous Community, and the need for coordination of these systems by the Spanish Government (with its Ministry of Health), which maintains the provision of health services in the Autonomous Cities of Ceuta and Melilla (despite their Statutes of Autonomy of 1995).

Sections in the Spanish Constitution of 1978 (SC) included the possibility that the Autonomous Communities (ACs) could assume competencies in health via transfer (section 148 and 150 of the EC). The General State Administration (State), for its part, would maintain the exclusive competence to establish the common bases and general coordination of healthcare throughout the Spanish territory (section 149 in SC), thus giving rise to an expansive, replicating and asymmetrical Public Sector [25].

The materialization of these provisions took place with the start of the decentralization process in health matters after the realization of the Autonomous Communities (1979–1981). This process was asymmetrical among the different regions, both in the type of competencies assumed and in the time at which they were transferred [26]. The so-called historical communities (Catalonia, the Basque Country and Galicia), plus Andalusia, were the first regions to assume sanitary powers. The need to complete decentralization in this area [27] led to the extension of healthcare competencies to all the ACs [28,29].

The nature of decentralization in health matters, which obeyed political rather than management and efficiency criteria [26], has led to the existence of a series of dysfunctions in the health system (manifesting itself in crises such as that of COVID-19) [30]. These include the problem of health financing and spending [31], the disparity between ACs’ health systems [31] and the need for greater coordination [29]. An example of this would be the inequality in access to health services among the ACs during the COVID-19 pandemic. This would be compounded by conflicts of competence in health matters between the national and regional levels of government [31].

However, one of its benefits would be the capacity to adapt to the territory and the needs of the population [27,28] and the increased capacity for transformation and innovation of the healthcare system [28,32]. In this way, decentralization would favor institutional change and improvement within the health system, as well as more specific attention to citizens’ demands. Consequently, addressing the Spanish health system implies taking into account each of the autonomous health systems, as well as the harmonizing role of the national government (to coordinate, not to centralize).

Taking all of this into consideration, the Spanish health system during the 2019–2020 period is evaluated. This evaluation is carried out through the analysis of four factors related to the management of the health system with respect to the pandemic. The period examined allows us to examine the consequences of health management before and during the COVID-19 pandemic, including the first two waves of it. The four factors analyzed are explained in detail below.

### 1.2. Health System Evaluation Factors

The bibliographic review carried out allows us to identify several important factors to evaluate the Spanish health system and its performance in the face of the COVID-19 pandemic. This is done once the starting situation has been identified, with a health system diminished by the deterioration of personal and economic resources and marked by the fragmentation of services generated by territorial decentralization. These elements of evaluation of the health system are: transparency, communication, reputation and well-being.

#### 1.2.1. Transparency in the Management of the Health System

Transparency is an indispensable element in the framework of democratic systems. Citizens must have access to all the information surrounding political decision-making and the functioning of public services, so that they can exercise effective control over the public authorities. However, information alone would not generate transparency, as it would require a complex process that would also depend on the context [33].

Transparency in the management of the health system is essential due to the need for accountability in an area as sensitive as health. In this sense, “transparency in the year of COVID-19 means tracking and publishing performance in the whole health system” [34] (p. 113). During the COVID-19 crisis, transparency has been demonstrated as a fundamental factor to fight against it [4]. The demand for transparency would have increased during the pandemic, as it was a confidence factor that would help reduce uncertainty [35]. Maintaining the trust of citizens in an emergency situation is essential. The adoption of difficult measures to manage the pandemic, and that affect citizens in a very negative way in different aspects, can only be accepted through transparency in that decision-making [36]. However, there are practically no studies that analyze comparatively the transparency of regional and national health systems during the pandemic [37]. This is especially important in decentralized countries, such as Spain, in which important decisions are made at different levels of government.

#### 1.2.2. Communication about the Management of the Health System

Communication about the health system allows the transmission of management information to citizens [38,39]. Likewise, communication by the health system would allow citizens to be given the necessary information on the measures to be adopted to prevent and stop infections. Collaboration with the media is essential in this task [2,3,4,5,6,7,8,9,10].

Furthermore, new technologies, especially the Internet, and not just direct personal experience, can promote such communication without the mediation of traditional media. During the COVID-19 pandemic, the use of digital technologies has intensified in many countries (e.g., South Korea, Taiwan, Israel, Australia, New Zealand) [14]. However, this new scenario is not without its dangers, since digital social networks promote greater exposure to negative information than traditional media, although it would also be present in these [40,41].

For this reason, it is important to assess whether the communication about the health system has been sufficient, on the one hand, and effective, on the other. This is justified by the importance of communication to stop the spread of the disease, something that should also be examined in the Spanish case.

#### 1.2.3. Reputation of the Health System

Reputation reflects the external image of any organization. Likewise, said reputation would reflect the management and provision of services carried out [4]. This reputation is shaped by the perception and evaluation of different stakeholders [10,42]. In this sense, a large part of the health reputation indicators are based on the evaluation of healthcare professionals and experts [42,43,44]. Hospitals are usually the most widely used health organizations to measure the reputation of the health system [42,43,44,45,46]. However, it is pointed out that not only should objective elements be considered, but also subjective elements such as those related to user perception [42]. In this regard, citizen evaluation would also be an important element of the efficiency of health systems [47]. For this reason, health reputation indicators have also been constructed based on the perception of citizens and patients [45]. In this sense, healthcare would be one of the public services best valued by citizens [31], despite its decentralization [21].

The reputation of the health system would be one of the main factors in managing the pandemic, by combining governance and legitimacy, which would favor effective communication [11]. Accordingly, health reputation cannot be examined without taking into account the decentralized nature of the Spanish healthcare system, since the characteristics of each Autonomous Community would condition health reputation [43].

#### 1.2.4. Well-Being Generated by the Health System

Due to the digital economy and in alignment with Horizon 2030, there is a shift from traditional welfare (social and material satisfaction) to well-being (personal and spiritual satisfaction also) [48,49]. This means that accountability is no longer heteronomous and limited to results (in accordance with state compliance regulations), but is becoming autonomous and people-oriented (companies are adopting internal codes for greater and better communication, participation and motivation of employees, and other stakeholders) [50]. Well-being evaluation seeks more satisfaction and it goes beyond hygienic measures to include motivational ones (for workers, citizens, etc.). This paradigmatic shift means focusing on personal well-being, something especially important in a context of exceptionality, also psychological, as the COVID-19 pandemic supposes. In this way, it is necessary to know the well-being generated by the management of the health system during the coronavirus crisis. Said well-being would be reflected in the satisfaction of citizens with said management, and not only in the satisfaction of healthcare professionals [51]. Therefore, it is of interest to examine the satisfaction of citizens with health management during this crisis, since the care received, or perceived, could make a difference in personal well-being. In this well-being generated by the health system, the importance of mental health stands out [52]. The well-being generated could vary in the different regions of the decentralized countries, as different health services are provided and received, as would happen in Spain [4].

### 1.3. Hypothesis

After examining the configuration of the Spanish health system and the factors to be used for its evaluation during the pandemic, the hypotheses are presented as possible answers to the research question: How has the Spanish health system managed the COVID-19 pandemic? The following hypotheses are formulated and will be empirically contrasted throughout the study:

**Hypothesis** **1.** *The health system has increased transparency during the COVID-19 pandemic.*

**Hypothesis** **2.** *The health system has increased the communication about the management during the COVID-19 pandemic.*

**Hypothesis** **3.** *The health system has increased its reputation during the COVID-19 pandemic.*

**Hypothesis** **4.** *The health system has increased the wellbeing, meaning satisfaction, generated by its management during the COVID-19 pandemic.*

## 2. Materials and Methods

The methodology used in this research follows a quantitative approach using both descriptive analysis and bi-variable analysis, which allow observing of the territorial differences among the variables for this study. The configuration of the Spanish health system requires the research to be carried out from a regional perspective, that is, to examine the different factors of health management in each of the autonomous health systems, including those of national government management when necessary. In line with this, and in accordance with the hypotheses put forward, four variables to be examined in the decentralized Spanish health system are identified.

The first variable is the transparency of the health system. This variable is measured by means of the transparency index of the autonomous health services carried out by Dynamic Transparency Index-Dyntra (applied to Spanish Healthcare system [53]. This indicator is made up of a total of 193 indicators divided into six groups: institutional transparency (53 indicators), public communication (20 indicators), citizen participation and collaboration (22 indicators), economic–financial transparency (25 indicators), service contracting (14 indicators), and healthcare transparency (59 indicators). These data will make it possible to examine the transparency of the regional healthcare systems before the arrival of the COVID-19 pandemic (in 2019).

Similarly, in order to analyze the transparency of the regional health systems during the management of the COVID-19 pandemic, as well as that of the central government’s management in this respect, the transparency index on COVID-19, also carried out by Dyntra, is used for health systems and its transparency [54]. This index (2020) is made up of 40 indicators distributed into four groups: transparency in health resources (10 indicators), transparency on infections (9 indicators), actions to mitigate the impact of COVID-19 (9 indicators) and economic transparency in the management of COVID-19 (12 indicators).

The second variable is communication about the management of the health system. The aim of this variable is to analyze how the flow of information about the health system has evolved with the arrival of the pandemic. The purpose of this is to observe whether or not the flow of information received by citizens about the health system and its management, normally the actors most distant from it, has increased after the irruption of COVID-19. This is particularly important, since communication capacity facilitates the transmission of information, which is essential for transparency, and contributes to shaping the perception of the different stakeholders, especially the public, about the different health systems and, consequently, their reputation, and ultimately, the well-being generated by the health management.

As indicators of the communication variable, CIS survey data are used on the media used by citizens to inform themselves about the pandemic (Study No. 3277) and on the average time of exposure to these media before and after the arrival of COVID-19 (Study No. 3305): (a) Study No. 3277 (March 2020): with a sample of 3911 interviews and a margin error of ±1.6% [55]; (b) Study No. 3305 (December 2020): a sample of 2084 interviews and a margin error of ±2.2% [56]. In addition to the information received through the media, citizens can shape their satisfaction with the health system by their direct experience with it. For this reason, the percentage of individuals in each Autonomous Community who required healthcare before and during COVID-19 is also included. The results are available in several polls: (a) Study No. 3281 (May 2020), with a sample of 3800 interviews and a margin error of ±1.6% [57]; (b) Study No. 3303 (December 2020), with a sample of 3817 interviews and a margin error of ±1.6% [58].

The third variable is the reputation of the health system. This variable is measured through two different and complementary perspectives. In this sense, the reputation of any organization, including healthcare organizations, is shaped by the perception of different stakeholders [21,31]. Health reputation, in line with this, has traditionally been measured through different indicators based on the perception of professionals and experts in the field.

Therefore, firstly, healthcare reputation is analyzed through Merco’s Healthcare Reputation Monitor-MRS (2019 edition and 2020 edition [59,60]. This indicator is constructed through the evaluation of various types of healthcare professionals (doctors, nurses and hospital pharmacists), patient associations, journalists specializing in healthcare and members of the healthcare administration. These actors evaluate different elements of the health system, including hospitals. In this way, they create a ranking of the 100 best hospitals in Spain. Based on this ranking, this research calculates a health reputation indicator that measures the percentage of hospitals out of the total number of existing hospitals that are included in the 100 most reputable hospitals in Spain. This is carried out in each of the autonomous health systems, thus making it possible to observe and compare the reputation of each of them prior to COVID-19 and during the pandemic.

Secondly, healthcare reputation is examined through the perception of citizens, who are the users and potential users of the health system. For this purpose, data from the Centro de Investigaciones Sociológicas (CIS) on the perception of Spanish citizens of the functioning of the healthcare system are used. Two CIS polls support this research: (a) Study No. 3259 (in October 2019, before the pandemic warning): sample of 2464 interviews and a margin error of ±2.0% [61]; (b) Study No. 3290 (in July 2020, during the pandemic second wave): sample of 2926 interviews and a margin error of ±1.8% [62]. In both polls, the main question was: In your opinion, how is the Healthcare system working? It works very, quite, little or not at all satisfactorily. In this sense, a comparison is made of the percentage of citizens who state that this functioning is very or fairly satisfactory in each Autonomous Community. This perception is compared before and during COVID-19, thus observing whether or not the reputation that citizens attribute to the health system in each region has changed with the arrival of the pandemic.

Finally, the fourth variable is well-being generated by the health management in three aspects: (a) health system, (b) management of the Spanish Government and (c) management of the Government of their respective Autonomous Community. A detailed analysis of the evolution of the satisfaction with these three aspects during the COVID-19 management itself is carried out, due to the fact that it has gone through different moments and phases. In this way, the aim is to discover how the satisfaction with the health management as perceived by the citizens of each Autonomous Community has varied throughout the pandemic. In this sense, we examine the percentage of individuals who declare that their satisfaction with the health system, with the management of the Spanish Government and with the management of the Government of their respective Autonomous Community, during the health crisis has improved, specifically between the first and second waves. Two CIS polls are used: (a) Study No. 3285 (June 2020): with a sample of 937 interviews and a margin error of ±3.3% [63]; (b) Study No. 3298 (October 2020): with a sample of 2861 interviews and a margin error of ±1.9% [64].

The data from the CIS surveys are perfectly comparable to each other, as shown by the multiple studies carried out with the data from this organization, since the CIS uses the same sample design and the same sampling procedure in all its surveys.

## 3. Results

The results of the analysis carried out to test the hypotheses and answer the research question are divided into four parts, according to the four factors by which the Spanish health system is evaluated. In this way, the results of transparency in the management of the health system, communication about the management of the health system, the reputation of the health system and the well-being generated by the health system are presented.

### 3.1. Transparency in the Management of the Health System during the COVID-19 Pandemic

**Figure 1:**
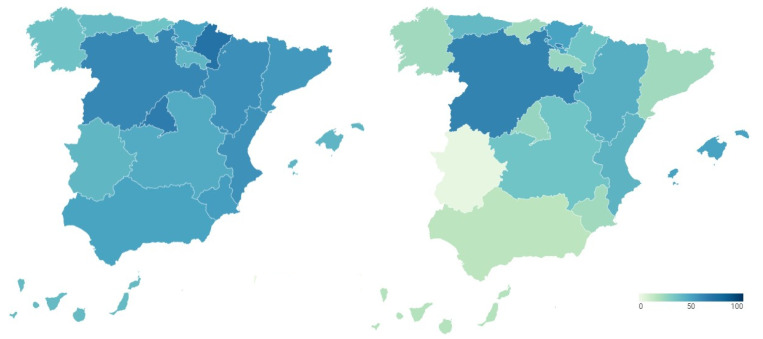
Transparency of Spanish regional health systems before and during COVID-19 (%).

To examine the transparency of health management during the COVID-19 pandemic, transparency indicators before and during the pandemic are analyzed and compared. In this way, Figure 1 presents the transparency data of the regional health systems before (map on the left) and during (map on the right) COVID-19. Detailed data can be consulted in Table A1 and Table A2 of Appendix A.

The transparency of the Spanish regional health systems before the outbreak of COVID-19 is shown in the map of the left of Figure 1. It shows the percentage of transparency achieved by the healthcare services of each Autonomous Community in the index carried out by Dyntra. This percentage represents the number of indicators that each autonomous health system complies with out of the total number of indicators that make up the index.

Navarre was the region with the highest level of healthcare transparency before the arrival of the new SARS-CoV-2 coronavirus (70.47%). It was followed by the Community of Madrid (65.28%) and Castilla y León (60.62%). With percentages above 50% were Aragón (57.51%), the Community of Valencia (56.48%), Catalonia (53.89%), the Region of Murcia (51.81%) and the Basque Country (50.78%). The healthcare systems of Andalusia (49.74%), Castilla-La Mancha (46.63%), the Balearic Islands (41.97%), La Rioja (41.97%), Extremadura (41.45%), Asturias (39.38%), the Canary Islands (39.38%), Galicia (36.27%) and Cantabria (35.75%) failed in transparency. These regional differences in the transparency of the health system, previous to the pandemic, are relevant.

The transparency in the management of COVID-19 by the different Autonomous Regions is shown in the map of the right of Figure 1. This figure shows the percentage of transparency achieved by the health management of the different Spanish regions during COVID-19 in the index carried out by Dyntra. This percentage represents the number of indicators that each Autonomous Community complies with out of the total number of indicators that make up the index.

Castilla y León is the Autonomous Community with the highest level of transparency in health management for COVID-19 (62.5%). It is followed by the Basque Country (50%) and the Balearic Islands (50%), these three being the only regions to pass in transparency during the pandemic. The rest of the Autonomous Regions fail in transparency, with some of them showing particularly low levels. In this sense, Aragón (45%), the Valencian Community (42.5%), Asturias (40%), Navarra (35%) and Castilla-La Mancha (35%) have percentages between 45% and 35%. With scores below 30% in health transparency are La Rioja (25%), the Community of Madrid (25%), the Region of Murcia (22.5%), Galicia (22.5%), Catalonia (22.5%) and Cantabria (22.5%). The Canary Islands (17.5%) and Andalusia (15%) do not reach 20%, while Extremadura shows the lowest level of transparency in COVID-19 management with only 2.5%. On the other hand, the Government of Spain also shows a low level of transparency in COVID-19 health management (27.5%). Again, there are regional differences in transparency into the health system, but this time, during the pandemic.

### 3.2. Communication about the Management of the Health System during the COVID-19 Pandemic

The information that citizens obtain about the health care system is conditioned by two elements: the image offered by the media and direct experience with it. Both factors contribute to shaping individuals’ image of the healthcare system and, consequently, their satisfaction with it. They are also the instruments through which the health system and its managers can transmit information on its functioning, thus increasing its transparency and reputation.

**Figure 2:**
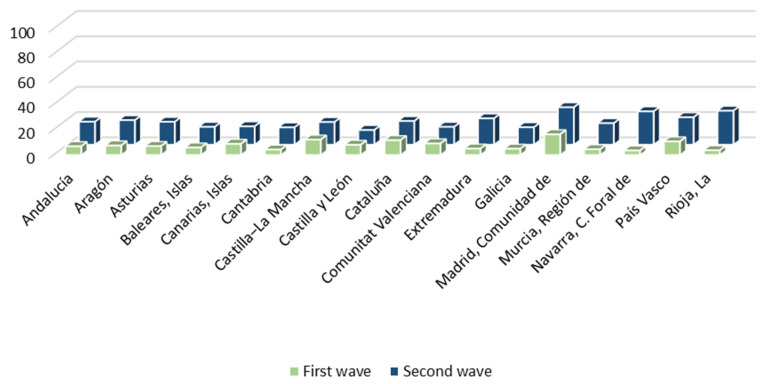
Citizens’ direct experience with healthcare services during the COVID-19 1st & 2nd wave (%).

As a consequence, both the direct experience of citizens with the health system and their exposure to the media during the pandemic are examined. Thus, Figure 2 shows the percentage of citizens who have visited the health services for symptoms related to the coronavirus during the first and second waves of the pandemic. These data are broken down by Autonomous Community. The proportion of citizens who have had a direct experience with health services does not exceed 30% in any of the pandemic waves considered. During the first wave of COVID-19, the citizens of the Community of Madrid (16.4%), Castilla-La Mancha (12.2%), Catalonia (11.8%) and the Basque Country (10.8%) were those who had to resort to the health system to the greatest extent, although they represent a very small percentage of the population. During the second wave, the Community of Madrid (29.6%), La Rioja (26.9%), Navarra (26.4%), the Basque Country (21.7%) and Extremadura (20.8%) were the regions that recorded the greatest contact of their citizens with health services, also representing limited proportions of the population. Consequently, the majority of citizens had no direct contact with the health system during the first two waves of the pandemic.

**Figure 3:**
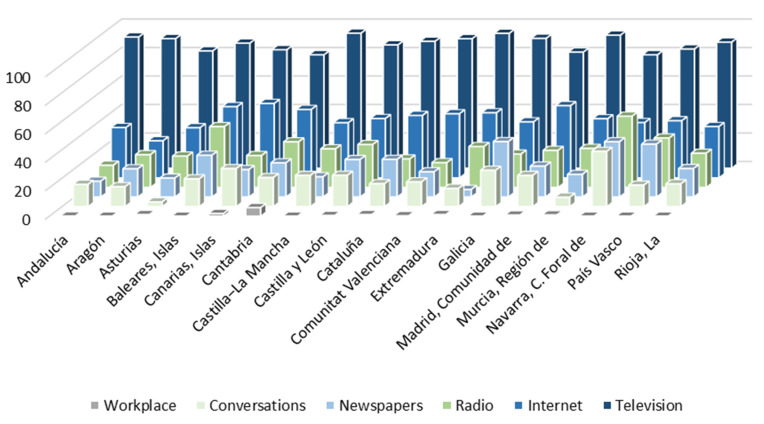
Media most used by citizens to obtain information on the COVID-19 pandemic (%).

This implies that a large part of the citizenship obtains information about the health system, and especially about the management of the pandemic, through the media. Thus, they would largely shape their perception of the health system and the management of COVID-19 through the media. In relation to this, more than 90% of Spanish have followed the news related to the coronavirus with some interest. Figure 3 shows the media through which they have followed the news about the pandemic. Television is configured as the main means of information for Spanish during the health crisis. More than eight out of ten citizens used it to follow COVID-19 news in all the ACs. Internet is the second most used channel, either through social networks, digital press or any other online media. Around four out of ten Spanish were informed about the pandemic through the Internet, with regions where this figure is even higher: the Canary Islands (52.2%), the Community of Madrid (50.7%) and the Balearic Islands (50%). Radio is the third most used means of communication. More than two out of ten Spanish have heard news about COVID-19 on the radio, with more than four out of ten in regions such as the Balearic Islands. Close behind is the written press, followed by obtaining information in conversations on the subject. The workplace, on the other hand, is a very residual medium for information on the pandemic.

**Figure 4:**
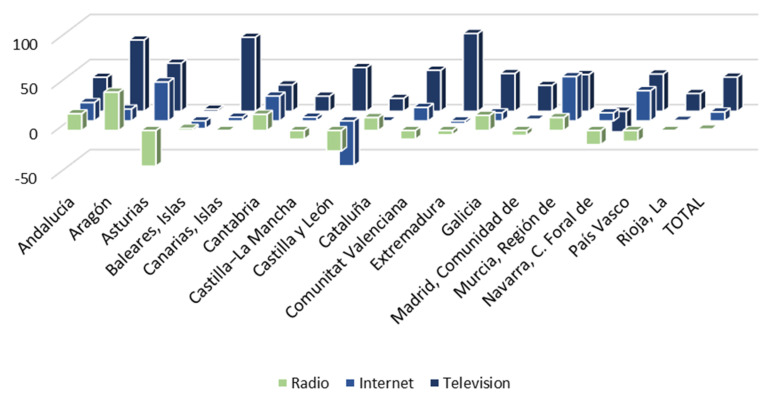
Average increase in time of exposure to the main media before and during COVID-19 (minutes).

Having identified the three media through which citizens have been most informed about the health crisis, it is then observed whether exposure to them has increased or not during the pandemic. As shown in Figure 4, the time of exposure to all media increased during the pandemic. Television is the medium whose use has increased the most. Thus, the Spanish watch 38.0 min more television per day on average than before the arrival of COVID-19. The use of social networks, specifically through the Internet, has also increased. Thus, citizens have increased by 9.9 min on average the daily time they spend connected to social networks. Likewise, although to a lesser extent, the time Spanish spend listening to the radio has also increased. Thus, they are exposed to this medium an average of 1.5 min more per day. Therefore, it is confirmed that the demand for information increases in times of health crisis, as there are relevant differences in media exposure after the arrival of the pandemic.

Taking into account regional differences, it is necessary to point out that the Autonomous Communities in which television consumption grew the most were Extremadura (86.4 min), the Canary Islands (82.2 min) and Aragon (79.2 min). The regions that most increased their exposure to the Internet were the Region of Murcia (49.2 min), Asturias (43.2 min) and the Basque Country (33.6 min). Finally, radio consumption increased especially in Aragon (42.6 min), Andalusia (18.6 min) and Cantabria (18.0 min).

However, despite the increase in average consumption of the media, some regions experienced decreases in some of them during the pandemic, especially in radio (Asturias, the Canary Islands, Castilla-La Mancha, Castilla y León, the Valencian Community, Extremadura, the Community of Madrid, Navarra and the Basque Country) and, to a lesser extent, on the Internet (the Balearic Islands, Castilla y León, Catalonia and Extremadura). Exposure to television, for its part, only decreased in Navarra, confirming this as the main medium through which citizens were informed of the health management of COVID-19.

### 3.3. Reputation of the Health System during the COVID-19 Pandemic

**Figure 5:**
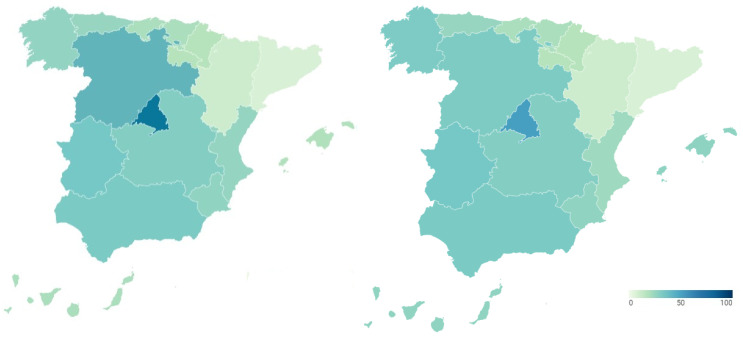
Reputation of Spanish regional health systems before and during COVID-19 according to the perception of health professionals & experts (%).

The reputation of the Spanish health system is measured from two perspectives. Firstly, the reputation of the health system is presented as a result of the perception of healthcare professionals and experts in the field. Figure 5 shows the percentage of hospitals in the top 100 of the Merco MRS out of the total number of hospitals that make up the healthcare system of each Autonomous Community before (map on the left) and during (map on the right) the pandemic. Detailed data can be consulted in Table A3 and Table A4 of Appendix A.

As can be seen in the map on the left, the Community of Madrid stands out above the rest of the Autonomous Communities in terms of health reputation, since 54.05% of its hospitals are among the top 100 hospitals in the ranking prepared by Merco before COVID-19. It is followed by Castilla y León (37.5%), Extremadura (33.33%), Andalusia (32%) and Castilla-La Mancha (30%). With levels below 30% in healthcare reputation are Murcia (26.67%), Galicia (26.32%), the Valencian Community (25.64%), Asturias (25%), the Canary Islands (20%), Cantabria (20%) and the Basque Country (20%). The regions with the lowest health care reputation are the Balearic Islands (18.18%), La Rioja (16.67%), Navarra (16.67%), Aragon (10%) and, especially, Catalonia (6.96%).

As can be seen in the map on the right, the reputation of the regional health systems, as a result of the perception of healthcare professionals and experts in the field, remains relatively stable with some changes in several Autonomous Communities. The Community of Madrid remains the region with the highest health reputation, although it experiences a slight drop in it to 51.35%. The Valencian Community and Castilla y León also experience a decline in the reputation of their health management during the pandemic, although of a moderate nature, falling to 23.08% and 31.25%, respectively. On the contrary, the Balearic Islands, the Canary Islands and Galicia register an increase in their health reputation during COVID-19, by increasing the percentage of hospitals that are among the Top 100 in the country. These percentages are 27.27%, 26.67% and 31.58%, respectively.

**Figure 6:**
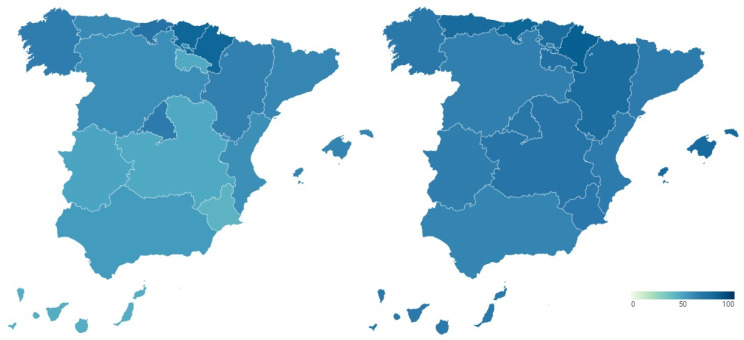
Reputation of Spanish regional health systems before and during COVID-19 according to citizen perception (%).

Secondly, we incorporate the health reputation resulting from the perception of Spanish citizens, who are the users and potential users of the health system. Figure 6 shows these data broken down again by Autonomous Community, showing the percentage of citizens who say that the functioning of the health system is very or fairly satisfactory in each of them before (map on the left) and during (map on the right) COVID-19. Detailed data can be consulted in Table A5 of Appendix A.

Before the pandemic, the Basque Country (81.5%) and Navarre (81.3%) are the regions with the best health reputation among their inhabitants. Cantabria (70.6%) also has a high level of citizen satisfaction with the functioning of its health system, as do the Community of Madrid (65.6%), Galicia (64.8%), Aragon (63.4%), the Balearic Islands (61.8%) and Catalonia (61.3%). Similarly, more than half the population of Asturias (59.7%), Castile and Leon (58.2%), the Valencian Community (57.3%) and Andalusia (53.1%) have a positive image of the health system in their Autonomous Community. In contrast, the health systems of Extremadura (49.2%), Castilla-La Mancha (47.3%), La Rioja (47.1%), the Canary Islands (45.3%) and the Region of Murcia (42.5%) show a lower reputation among their citizens, although with not particularly low figures.

During the pandemic, the data show that the health crisis generated by the new SARS-CoV-2 coronavirus led to an increase in the reputation of the health system, according to the citizens’ perspective, in all the Autonomous Communities four months after its outbreak. Thus, the percentage of citizens declaring themselves to be very or fairly satisfied with the functioning of the health system is over 60% in all regions. The health systems of Navarre (84.6%) and Cantabria (82.1%) have the highest reputation among the population. They are followed by the Balearic Islands (78.1%), Asturias (77.4%), the Basque Country (76.6%), Aragon (75.9%) and La Rioja (73.3%). Lastly, we find Castilla-La Mancha (68.9%), the Community of Madrid (68.9%), Galicia (67.2%), the Region of Murcia (67.1%), the Canary Islands (66.9%), the Community of Valencia (65.8%), Catalonia (65.7%), Extremadura (64.4%), Castilla y León (63.7%) and Andalusia (61.7%). These data imply that there are no significant differences between the reputations that citizens attribute to the health systems of the different Autonomous Regions.

### 3.4. Well-Being Generated by the Health System during COVID-19 Pandemic

Well-being generated by the health management is an essential issue, but it is especially so in a pandemic situation, due to the exceptional nature, also psychological, that it entails for all stakeholders. However, the management of the COVID-19 pandemic has gone through different moments and phases, so that citizens’ perception of it may have varied accordingly, rather than being configured as a fixed image. In line with this, the following examines the satisfaction of citizens at two different moments of the health crisis on three different aspects: the health system, the management of the Spanish government and the management of the government of their respective Autonomous Community. The percentage of citizens who recognize that their satisfaction with each of these has improved during the first wave (June 2020) and the second wave (October 2020) of the pandemic is represented.

**Figure 7:**
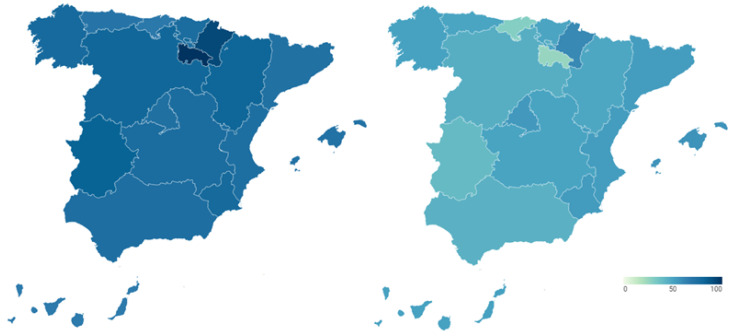
Citizen satisfaction with the health system during the COVID-19 1st & 2nd wave (%).

Figure 7 presents the data on the improvement in citizens’ satisfaction with the health care system during the first (map on the left) and second (map on the right) waves of COVID-19. Detailed data can be consulted in Table A6 of Appendix A. As can be seen visually, the percentage of citizens indicating that their opinion of the health system has improved decreases over time. This means that citizen satisfaction with the health system has worsened over the course of the pandemic, despite being very positive at the beginning of the pandemic. La Rioja (75 percentage points) and Extremadura (43.9 percentage points) are the Autonomous Regions in which the proportion of citizens declaring that their satisfaction with the health system has improved has fallen the most. Next come Cantabria (36.9 points), Castile and Leon (35.7 points), Aragon (33.3 points), Navarre (32.3 points) and Andalusia (31.2 points). With decreases of less than 30 percentage points are Galicia (27.4 points), the Basque Country (27.2 points), Castilla-La Mancha (26.6 points), the Region of Murcia (25.6 points), the Community of Valencia (21.9 points), Catalonia (20.4 points) and Asturias (20.3 points). The regions in which the satisfaction with the healthcare system has deteriorated the least are the Canary Islands (15 points), the Balearic Islands (15.4 points) and the Community of Madrid (19.7 points), in that order.

**Figure 8:**
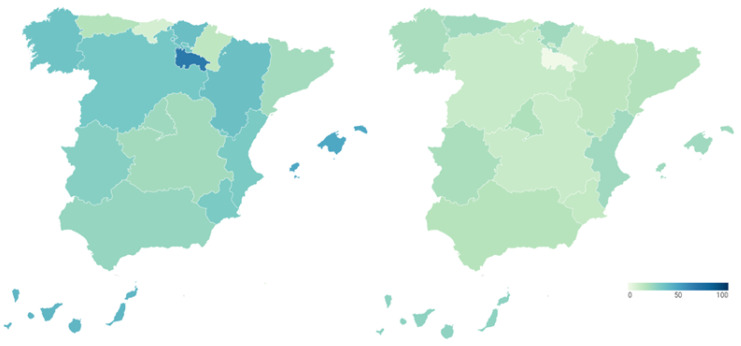
Citizen satisfaction with the management of the Spanish government during the COVID-19 1st & 2nd wave (%).

Figure 8 shows the information on the improvement of citizen’s satisfaction with the management of the Spanish government during the first (map on the left) and second (map on the right) waves of the pandemic. Detailed data can be consulted in Table A6 of Appendix A. As with the health care system, the percentage of Spanish who say that their satisfaction with the national government has improved decreases throughout the health crisis. Thus, the satisfaction with the central government and its management of COVID-19 becomes more negative as time passes. However, the perception of the country’s government was negative from the beginning of the pandemic, since only a small part of the population in most of the Autonomous Communities improved their satisfaction with it. Thus, the majority of citizens worsened their satisfaction of the central executive with the arrival of the COVID-19 pandemic. This negative perception also increased as the pandemic progressed.

The percentage of citizens who reported an improvement in their satisfaction with the Spanish government at the beginning of the health crisis did not exceed 40% in any region, with the exceptions of the Canary Islands (41.5%), the Balearic Islands (47.6%) and La Rioja (66.7%). This perception also worsened with the arrival of the second wave, with La Rioja, the Balearic Islands, Aragón and Castilla y León being the Autonomous Regions most affected, with a drop of 66.7, 24.9, 22.2 and 21.6 percentage points, respectively. They are followed by the citizens of the Region of Murcia (18.9 points), Galicia (15.2 points), the Basque Country (15.2 points), the Canary Islands (14.3 points) and Castilla-La Mancha (10.3 points). Smaller differences exist between users in Extremadura (9.4 points), the Community of Valencia (9.3 points), Andalusia (8.9 points), the Community of Madrid (4.6 points), Navarre (4.3 points) and Catalonia (3.7 points). On the other hand, in Asturias and Cantabria the percentage of citizens who improve their satisfaction with the national government increased by 5.1 and 5.2 percentage points (each one).

**Figure 9:**
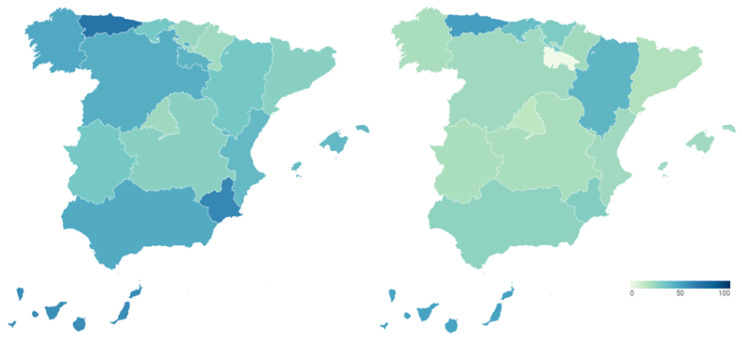
Citizen satisfaction with the management of the government of their respective Autonomous Community during the COVID-19 1st & 2nd wave (%).

Finally, Figure 9 shows the data on the improvement of citizen satisfaction with management of the government of their respective Autonomous Community during the first (map on the left) and second (map on the right) waves of the pandemic. Detailed data can be consulted in Table A6 of Appendix A. In the initial phase of the pandemic, there were disparities among citizens regarding the perception of their respective regional governments. Thus, while in Asturias, 69.6% of the population had improved their satisfaction with the regional executive, in Navarre, this figure was only 23.1%. However, despite these differences, most of the Autonomous Regions experienced a worsening of citizen perception of the regional government throughout the pandemic, although in many cases not excessively high. Thus, this deterioration is more important in the Region of Murcia, amounting to 30.6 percentage points. It is followed by Castilla y León (22.3 points), Galicia (20 points), Andalusia (19.3 points), Asturias (17.5 points), the Balearic Islands (17.3 points) and Catalonia (10.2 points). In the Community of Madrid (8.8 points), the Canary Islands (8.5 points), Castilla-La Mancha (8.4 points), Extremadura (6.6 points) and La Rioja (5.4 points), on the other hand, the change of opinion is smaller. Likewise, in Navarra (0.6 points) and in the Valencian Community (0.2 points), the satisfaction with the regional executive remained practically stable throughout the pandemic. On the other hand, the citizens of Cantabria, the Basque Country and Aragon improved their perception of the regional government between the first and second wave of the pandemic, with 4.5%, 6.4% and 8.6% of them doing so. Thus, we can see that there are regional differences in the citizen satisfaction with their respective regional executives.

## 4. Discussion

This research has evaluated how the Spanish health system has managed the COVID-19 pandemic. This is done by analyzing four key factors of health management before and during the arrival of the new SARS-CoV-2 coronavirus: transparency in the management of the health system, communication about the management of the health system, the reputation of the health system and the well-being generated by the health system. Due to the decentralized nature of the Spanish health system, this analysis is carried out by disaggregating the data by Autonomous Community [24,25].

The results show that the health crisis triggered by the arrival of the COVID-19 pandemic has had a considerable impact on health management, producing changes in all the factors analyzed.

In general terms, it can be stated, firstly, that there has been deteriorated in the transparency of the Spanish health system during the health crisis. From a situation of relatively acceptable transparency in most regions, although with significant differences between them, the situation has shifted to one of greater opacity. Public information on the management of the pandemic is limited in all the Autonomous Regions, as well as on the part of the national government, increasing considerably the disparities between regions. In this sense, the public authorities have had to transform their communication policy, based on traditional information on health services, to a totally different reality, that of a new and unknown pandemic disease, having to do so in conditions of health emergency, which has shown a significant deficiency in respect of such management. This lack of transparency could also be explained by the absence of a clear distribution of functions and responsibilities among the different levels of government, that is, national and regional. Deficiencies in transparency do not allow the generation of trust among the population in a context of uncertainty. Hypothesis 1 is therefore rejected. These results contrast with the importance of transparency in pandemic management, as has been demonstrated in other countries [4,5,6,7,8,9,10,11,12,13,14,15,16,17,18,19,20,21,22,23,24,25,26,27,28,29,30,31,32,33,34,35,36].

Secondly, with regard to communication, there has been an increase in the flow of health information with the advent of COVID-19. The vast majority of citizens have followed the news about the pandemic with interest. Likewise, there has been an increase in the amount of time that citizens have been exposed to the most widely used media to learn about the health crisis. This implies that information on health and its management is not only more in demand, but also floods the media. Therefore, the visibility of health management has increased enormously. In this way, Hypothesis 2 is accepted. Nevertheless, the fact that most citizens have not had direct contact with the health system means that they have access to information, which is essential for transparency, shaping their perception of the health system, and consequently the reputation of the health system, through the media. This may help explain the deterioration in personal well-being generated by the health management during the pandemic because there could have occurred a problem of perception, infoxication (information overload) or disinformation, especially with the use of the Internet. Likewise, the absence of a joint communication strategy between health institutions and the media, which has existed in other countries, makes it possible that, although exposure increases, information regarding the health system may be more negative. In this way, the lack of collaboration between the different actors would make it difficult to fight the pandemic. In summary, these results suggest that the flow of communication about the health system has increased, in accordance with its importance in a health crisis [2,3,4,5,6,7,8,9,10,11,12,13,14,15,16,17,18,19,20,21,22,23,24,25,26,27,28,29,30,31,32,33,34,35,36,37,38,39]. However, there has been no collaboration for the implementation of a communication strategy between health authorities and the media, something essential in the success of management in other countries [10,11,12,13,14,15,16,17].

Thirdly, the reputation of the health system is shaped by the perception of various stakeholders [21,22,23,24,25,26,27,28,29,30,31]. This article has analyzed it from the perspective of professionals and experts in the field, on the one hand, and from the perspective of citizens, on the other. The health reputation from the perspective of health professionals and experts shows the existence of great divergences between Autonomous Regions before the arrival of the pandemic. With the arrival of the health crisis, the data on health reputation from this perspective doesn’t show important changes. Some regions slightly worsen their health reputation, while others improve it moderately. In general terms, the reputation of the healthcare system according to the perception of healthcare professionals and experts shows no notable differences during the healthcare crisis, with regional disparities remaining.

Health reputation from the citizen’s perspective, on the other hand, presents more positive data. The initial regional differences narrow until they practically disappear during COVID-19, as homogeneity in the perception of regional health care systems increases. In this way, the evaluation of the management of the health system during the pandemic and of the provision of health services is positive on the part of citizens, with a positive image. In relation to this, the arrival of the pandemic brought with it an increase in the reputation of the health system from the public’s perspective. For this reason, Hypothesis 3 is accepted. These results suggest that the evaluation of the management of the health system has led to an increase in its reputation from the citizen perspective [41,42,43,44,45,46], which may favor legitimacy [11].

Fourth, the subsequent development of the pandemic led to a deterioration in the well-being generated by the health system during COVID-19. The loss of well-being is detected in the three indicators evaluated and it is generalized in all regions, although satisfaction levels vary among them, also starting from different levels. In relation to this, although the levels of satisfaction in the regions are different, since the health services provided are also different in a decentralized country, there is a common tendency to decrease the well-being generated by the health system. Consequently, Hypothesis 4 is also rejected. This deterioration occurs especially with respect to the health management of the national government and, after it, with respect to the health management of the governments of the Autonomous Communities. In this way, the management of the national government would be the one that would generate the least well-being, followed by the management of the autonomous government. The healthcare system would be the one that would generate greater well-being, a result that would be in line with the increase in its reputation among citizens, despite its progressive decline with the advance of the pandemic. These results reflect the difficulty of the Spanish health system to attend to the well-being of citizens [4], despite its great importance in a context of pandemic [51,52]. This translates into a deterioration in the population’s satisfaction with health management, regardless of the level of government [4].

### 4.1. Implications

Previous studies on health systems and their different elements have been carried out in a context of normality. However, the outbreak of a pandemic such as the one that occurred with COVID-19 poses a major challenge for research in this area. The health systems are on the front line in the fight against the new SARS-CoV-2 coronavirus, so it is essential to examine how the pandemic has affected them, identifying which are the factors that contribute to a successful management against COVID-19 and its consequences and evaluating these factors in the different health systems. Several studies have started this task, making it necessary to continue the analysis of the factors that determine the failure or success in managing the pandemic in different national contexts. In the present study, this is also in a case such as Spain, which has been one of the European countries most affected by the pandemic (as has been officially recognized). This analysis not only addresses the perspective of professionals and experts in the field, but also the citizen’s perspective, which is unavoidable in this scenario. Therefore, the relationship of citizens with the health system and its managers at different levels of government is taken into consideration.

This research is one more step in the study of the effects of COVID-19 on health system and health management, reaching important conclusions. The information obtained may be valid not only for accountability, but also for decision making by public authorities regarding the different factors evaluated. The analysis of the factors that have contributed to the failure and success in the fight against the pandemic can contribute to improving the preparedness of the health system and health management for future health emergencies, which could be related, for example, to climate change.

### 4.2. Research Limitations and Future Lines

This research has several limitations derived from the timing of its implementation. In this regard, by evaluating the Spanish health system during COVID-19 while still in the midst of the pandemic, we do not yet have a sufficient amount of data to allow a more in-depth analysis of the four factors of health management under investigation: transparency, communication, reputation and well-being. Likewise, with respect to the study period, we have only been able to investigate the consequences of the COVID-19 pandemic on the Spanish health system during the first two waves of the pandemic.

Nevertheless, this research is a first approach that may contribute to future research to advance the study of the Spanish health system, and in general, of the health systems, once the pandemic is over. In this way, broad conclusions can be reached about the factors that have contributed to the failure or success of health systems in the face of COVID-19. In this respect, the approach adopted in this research, disaggregating the data by region due to the decentralized nature of the Spanish health system, may prove useful.

The impact of the pandemic on accelerating digitization remains to be studied in depth. The importance of new technologies in the management of the pandemic at all levels is a line of research to be developed. Detecting the strengths and weaknesses of the digital tools used in the management of the health system can allow the development of new strategies for their use in future health crises.

## 5. Conclusions

The health system is at the center of managing the pandemic. With COVID-19, there was a double management problem: a health administrative problem and a health perception problem. The more information was provided to the citizens, the worse satisfaction was obtained by the health system, as a problem of perception and infoxication (information overload). Additionally, this study shows that the health management and its perception by the social actors have changed with the progress of the pandemic crisis. Possibly, the situation has been complicated by the decentralized nature of the Spanish healthcare system, as a large part of healthcare competencies has been transferred to the ACs. In this respect, despite the enormous increase in the flow of communication about the health system and its management, information transparency on the part of the ACs and the national government has not been able to adapt to this new scenario throughout the country. The reputation of the health system, for its part, seems to have increased at the beginning of the pandemic in a climate of unity. However, from the public’s perspective, as the flow of information is received, the satisfaction of the health system is deteriorating, given the apparent lack of coordination between the national and regional management of the pandemic (because of the game of power between the Administrations levels).

The problem goes beyond the question of accountability and wellbeing evaluation, since the development of a crisis such as that of COVID-19 has led to the quality and reliability of the Spanish health system being called into question: material insufficiencies (beds, masks, PPE, etc.), lack of common management and solidarity between ACs (lack of coordination in health measures adopted, failure to temporarily transfer equipment, etc.). It even seems that the advances achieved in health management of well-being (more satisfaction and better relations between people/planet/profit) have been renounced, losing attention to improve the satisfaction and the motivational measures. This is a key point, because the COVID-19 crisis is not just a pandemic, it is also a syndemic, which affects material and psychological issues (a topic for future lines of research).

## Data Availability

Not applicable.

## Appendix A

**Table A1:** Transparency of Spanish regional healthcare systems before COVID-19 (percentage/indicators).

	TOTAL	Institutional Transparency	Public Communication	Participation/Citizen collaboration	Economic-Financial Transparency	Contracting of Services	Health transParency
Navarra, C. Foral de	70.47% (136/193)	73.58% (39/53)	90.00% (18/20)	63.64% (14/22)	64.00% (16/25)	64.28% (9/14)	67.80% (40/59)
Madrid, Comunidad de	65.28% (126/193)	71.70% (38/53)	70.00% (14/20)	54.55% (12/22)	40.00% (10/25)	57.14% (8/14)	74.58% (44/59)
Castilla y León	60.62% (117/193)	60.38% (32/53)	75.00% (15/20)	63.64% (14/22)	32.00% (8/25)	78.57% (11/14)	62.71% (37/59)
Aragón	57.51% (111/193)	60.38% (32/53)	65.00% (13/20)	36.36% (8/22)	24.00% (6/25)	85.71% (12/14)	67.80% (40/59)
Comunitat Valenciana	56.48% (109/193)	52.83% (28/53)	70.00% (14/20)	59.09% (13/22)	44.00% (11/25)	71.43% (10/14)	55.93% (33/59)
Cataluña	53.89% (104/193)	56.6% (30/53)	80.00% (16/20)	63.64% (14/22)	48.00% (12/25)	42.86% (6/14)	44.07% (26/59)
Murcia, Región de	51.81% (100/193)	52.83% (28/53)	60.00% (12/20)	50.00% (11/22)	64.00% (16/25)	71.43% (10/14)	38.98% (23/59)
País Vasco	50.78% (98/193)	56.60% (30/53)	80.00% (16/20)	72.73% (16/22)	32.00% (8/25)	64.29% (9/14)	32.20% (19/59)
Andalucía	49.74% (96/193)	58.49% (31/53)	75.00% (15/20)	59.09% (13/22)	24.00% (6/25)	71.43% (10/14)	35.59% (21/59)
Castilla-La Mancha	46.63% (90/193)	60.38% (32/53)	75.00% (15/20)	59.09% (13/22)	28.00% (7/25)	71.43% (10/14)	22.03% (13/59)
Baleares, Islas	41.97% (81/193)	54.72% (29/53)	60.00% (12/20)	59.09% (13/22)	40.00% (10/25)	42.86% (6/14)	18.64% (11/59)
La Rioja	41.97% (81/193)	50.94% (27/53)	70.00% (14/20)	54.55% (12/22)	36.00% (9/25)	50.00% (7/14)	20.34% (12/59)
Extremadura	41.45% (80/193)	54.72% (29/53)	65.00% (13/20)	31.82% (7/22)	36.00 (9/25)	64.29% (9/14)	22.03% (13/59)
Asturias	39.38% (76/193)	43.4% (23/53)	65.00% (13/20)	50.00% (11/22)	24.00% (6/25)	64.29% (9/14)	22.73% (14/59)
Canarias, Islas	39.38% (76/193)	49.06% (26/53)	70.00% (14/20)	54.55% (12/22)	24.00% (6/25)	64.29% (9/14)	15.25% (9/59)
Galicia	36.27% (70/193)	47.17% (25/53)	65.00% (13/20)	45.45% (10/22)	28.00% (7/25)	64.29% (9/14)	10.17% (6/59)
Cantabria	35.75% (69/193)	50.94% (27/53)	45.00% (9/20)	36.36% (8/22)	36.00% (9/25)	64.29% (9/14)	11.86% 7/59)

**Table A2:** Transparency of Spanish regional healthcare systems during COVID-19 (percentage/indicators).

	TOTAL	Transparency in Health Resources	Transparency About Infections	Actions to Alleviate COVID-19	Economic Transparency in Management
Castilla y León	62.50% (25/40)	90.00% (9/10)	100.00% (9/9)	44.44% (4/9)	25.00% (3/12)
País Vasco	50.00% (20/40)	20.00% (2/10)	66.67% (6/9)	77.78% (7/9)	41.67% (5/12)
Baleares, Islas	50.00% (20/40)	30.00% (3/10)	66.67% (6/9)	55.56% (5/9)	50.00% (6/12)
Aragón	45.00% (18/40)	20.00% (2/10)	100.00% (9/9)	44.44% (4/9)	25.00% (3/12)
Comunitat Valenciana	42.50% (17/40)	20.00% (2/10)	66.67% (6/9)	77.78% (7/9)	16.67% (2/12)
Asturias	40.00% (16/40)	30.00% (3/10)	55.56% (5/9)	44.44% (4/9)	33.33% (4/12)
Castilla-La Mancha	40.00% (16/40)	10.00% (1/10)	55.56% (5/9)	66.67% (6/9)	33.33% (4/12)
Navarra, C. Foral de	35.00% (14/40)	20.00% (2/10)	77.78% (7/9)	22.22% (2/9)	25.00% (3/12)
Gobierno de España	27.50% (11/40)	10.00% (1/10)	66.67% (6/9)	44.44% (4/9)	0.00% (0/12)
La Rioja	25.00% (10/40)	10.00% (1/10)	66.67% (6/9)	33.33% (3/9)	0.00% (0/12)
Madrid, Comunidad de	25.00% (10/40)	10.00% (1/10)	44.44% (4/9)	33.33% (3/9)	16.67% (2/12)
Murcia, Región de	22.50% (9/40)	10.00% (1/10)	66.67% (6/9)	22.22% (2/9)	0.00% (0/12)
Galicia	22.50% (9/40)	20.00% (2/10)	22.22% (2/9)	33.33% (3/9)	16.67% (2/12)
Cataluña	22.50% (9/40)	10.00% (1/10)	55.56% (5/9)	33.33% (3/9)	0.00% (0/12)
Cantabria	22.50% (9/40)	20.00% (2/10)	55.56% (5/9)	22.22% (2/9)	0.00% (0/12)
Canarias, Islas	17.50% (7/40)	10.00% (1/10)	55.56% (5/9)	11.11% (1/9)	0.00% (0/12)
Andalucía	15.00% (6/40)	10.00% (1/10)	44.44% (4/9)	11.11% (1/9)	0.00% (0/12)
Extremadura	2.50% (1/40)	0.00% (0/10)	11.11% (1/9)	0.00% (0/9)	0.00% (0/12)

**Table A3:** Reputation of Spanish regional healthcare systems before COVID-19 according to the perception of health professionals & experts.

	Number of Hospitals in Top-100	Total Number of Hospitals	Percentage of Hospitals in Top-100	Percentage of Hospitals Over the National Total
Andalucía	16	50	32.00	10.68
Aragón	2	20	10.00	4.27
Asturias	3	12	25.00	2.56
Baleares, Islas	2	11	18.18	2.35
Comunitat Valenciana	10	39	25.64	8.33
Canarias, Islas	3	15	20.00	3.21
Cantabria	1	5	20.00	1.07
Castilla-La Mancha	6	20	30.00	4.27
Castilla y León	6	16	37.50	3.42
Cataluña	11	158	6.96	33.76
Extremadura	4	12	33.33	2.56
Galicia	5	19	26.32	4.06
La Rioja	1	6	16.67	1.28
Madrid, Comunidad de	20	37	54.05	7.91
Murcia, Región de	4	15	26.67	3.21
Navarra, C. Foral de	1	6	16.67	1.28
País Vasco	5	25	20.00	5.34
TOTAL	100	466		

**Table A4:** Reputation of Spanish regional healthcare systems during COVID-19 according to the perception of health professionals & experts.

	Number of Hospitals in Top 100	Total Number of Hospitals	Percentage of Hospitals in Top 100	Percentage of Hospitals Over the National Total
Andalucía	16	50	32.00	10.68
Aragón	2	20	10.00	4.27
Asturias	3	12	25.00	2.56
Baleares, Islas	3	11	27.27	2.35
Comunitat Valenciana	9	39	23.08	8.33
Canarias, Islas	4	15	26.67	3.21
Cantabria	1	5	20.00	1.07
Castilla-La Mancha	6	20	30.00	4.27
Castilla y León	5	16	31.25	3.42
Cataluña	11	158	6.96	33.76
Extremadura	4	12	33.33	2.56
Galicia	6	19	31.58	4.06
La Rioja	1	6	16.67	1.28
Madrid, Comunidad de	19	37	51.35	7.91
Murcia, Región de	4	15	26.67	3.21
Navarra, C. Foral de	1	6	16.67	1.28
País Vasco	5	25	20.00	5.34
TOTAL	100	466		

**Table A5:** Reputation of Spanish regional health systems before and during COVID-19 according to citizen perception (% of citizens who say that the functioning of the healthcare system is very or fairly satisfactory).

	Before COVID-19	During COVID-19
Andalucía	53.1	61.7
Aragón	63.4	75.9
Asturias	59.7	77.4
Baleares, Islas	61.8	78.1
Comunitat Valenciana	57.3	65.8
Canarias, Islas	45.3	66.9
Cantabria	70.6	82.1
Castilla-La Mancha	47.3	68.9
Castilla y León	58.2	63.7
Cataluña	61.3	65.7
Extremadura	49.2	64.4
Galicia	64.8	67.2
La Rioja	47.1	73.3
Madrid, Comunidad de	65.6	68.9
Murcia, Región de	42.5	67.1
Navarra, C. Foral de	81.3	84.6
País Vasco	81.5	76.6

**Table A6:** Citizen satisfaction with the health system, the management of the Spanish government and the management of the government of their respective Autonomous Community during the COVID-19 1st & 2nd wave (%).

	Health System		Spanish Government		Autonomous Government	
	First Wave	Second Wave	First Wave	Second Wave	First Wave	Second Wave
Andalucía	74.7	43.6	25.3	16.4	46.5	27.1
Aragón	81.5	48.1	37.0	14.8	33.3	42.0
Asturias	69.6	49.3	17.4	22.5	69.6	52.1
Baleares, Islas	71.4	56.1	47.6	22.7	40.0	22.7
Comunitat Valenciana	74.2	52.3	32.0	22.7	40.2	40.0
Canarias, Islas	65.9	50.9	41.5	27.2	58.5	50.0
Cantabria	66.7	29.7	8.3	13.5	33.3	37.8
Castilla-La Mancha	76.2	49.6	22.0	11.6	28.6	20.2
Castilla y León	80.8	45.1	33.3	11.7	45.1	22.8
Cataluña	72.8	52.4	21.6	17.9	28.6	18.4
Extremadura	83.3	39.4	29.2	19.7	33.3	26.8
Galicia	78.0	50.5	35.6	20.4	47.5	27.4
La Rioja	100.0	25.0	66.7	0.0	42.9	37.5
Madrid, Comunidad de	73.4	53.7	23.4	18.8	23.4	14.6
Murcia, Región de	78.6	53.0	32.1	13.3	60.7	30.1
Navarra, C. Foral de	92.3	60.0	14.3	10.0	23.1	22.5
País Vasco	76.1	48.9	37.8	22.6	25.0	31.4
